# Parental Tetracycline Exposure Induces Genotoxic Damage and Fitness Deficits in *Daphnia magna* Offspring

**DOI:** 10.1002/em.70077

**Published:** 2026-08-22

**Authors:** Buse Ceyda Öncel, Çiğdem Akın Pekşen, Beste Tunca, Zeynep Kavasoğlu, Özlem Darcansoy İşeri

**Affiliations:** ^1^ Department of Molecular Biology and Genetics, Faculty of Science and Letters Başkent University Ankara Türkiye; ^2^ Institute of Transplantation and Gene Sciences, Başkent University Ankara Türkiye; ^3^ Institute of Food, Agriculture and Livestock Development, Başkent University Ankara Türkiye

**Keywords:** antibiotic contamination, aquatic model organism, DNA damage, individual fitness, ONE Health, population growth, water fleas

## Abstract

Antibiotics contaminating aquatic environments is a challenge for the ONE Health initiative as it poses a risk to biodiversity. Tetracyclines are toxic to a wide range of organisms. The objective of this study was to assess the growth, individual fitness and DNA damage following a short‐term exposure to tetracycline in *Daphnia magna* . Additionally, the long‐term effects of DNA damage on population growth rate and individual fitness parameters in the offspring for intergenerations were evaluated. The results indicated that *D. magna* has a significant effect on tetracycline‐induced fertility impairment, resulting in reduced survival and reproductive capacity of the parental groups and impaired growth and development of the offspring. Furthermore, tetracycline treatment resulted in a significant reduction in individual fitness, as evidenced by the significant reduction in maximum body length of *D. magna* in both tetracycline‐exposed generations. *D. magna* was exposed to concentrations of 25, 50, 100, 200, and 400 mg/L for periods of 24–72 h, with the objective of determining the concentrations that would not be lethal and yet would induce DNA damage. Tetracycline concentrations of 50 and 25 mg/L did not result in excessive levels of cytotoxicity within 72 h. P, F1, and F1 offspring born between 48 and 72 h were tested for DNA damage. *D. magna* appears to recover from DNA damage, but concerns have been raised about using tetracycline repeatedly in water, as it can lead to higher concentrations and longer exposure times for lots of organisms.

**Abbreviations**
AU: arbitrary units
EC_50_
: half‐maximal effective concentration
F1: first generation
F2: second generation
F3: third generation
LMPA: low melting point agarose
n1: F1, born between 0 and 24 h
n2: F1, born between 24 and 48 h
n3: F1, born between 48 and 72 h
NMPA: normal melting point agarose
OECD: Organization for Economic Co‐operation and Development
P: parent generation
SCGE: single cell gel electrophoresis
TC: tetracycline

## 1 Introduction

Tetracyclines (TC), including tetracycline, oxytetracycline, and chlortetracycline, are bacteriostatic antibiotics that were used in broad‐spectrum for the treatment of aerobic gram‐positive and gram‐negative bacterial infections. They interact with the 16S ribosomal RNA of the bacterial 30S ribosomal subunit, resulting in the inhibition of the insertion of aminoacyl‐transfer RNA. This, in turn, leads to the interruption of translation. Furthermore, they have been demonstrated to induce conformational alterations, deletions, and diverse mutations in ribosomal proteins (Grossman et al. 2012; Trieber and Taylor 2002). Therefore, tetracyclines have been widely used in human medicine, veterinary medicine, and aquaculture for approximately 70 years (Belkheiri et al. 2011). However, TCs exert toxic effects on plants, sewage sludge bacteria (Halling Sorensen et al. 1998), and a multitude of other organisms that are not the intended target, such as *Daphnia magna* (water fleas), which live in aquatic environments (Wollenberger et al. 2000). Particularly, tetracyclines exhibit a strong affinity for clay materials, soil, and sediments due to the presence of multiple electron‐rich functional groups, including ketone, amide, and hydroxyl groups. These functional groups facilitate the formation of polyvalent cations and stable metal–ligand complexes, which contribute to the strong affinity of tetracyclines for these natural materials. This affinity has been attributed to the high degree of tetracycline absorption exhibited by these natural materials. Therefore, tetracyclines are regarded as one of the most significant ecotoxic pollutants (Halling Sorensen et al. 1998; Wollenberger et al. 2000). The objective of ONE Health is to ensure the sustainable protection of the health of humans, animals, plants, and the environment through a holistic approach. Contaminating soil and aquatic environments with antibiotics poses a significant challenge to the ONE Health initiative. This contamination endangers biodiversity and may contribute to the emergence of antibiotic‐resistant pathogenic bacteria.

In addition to their nontargeted impact on the environment, tetracyclines can lead to different degrees of damage at both cellular and genome level. For example, they cause phototoxic reactions in the skin, resulting in the production of metastable quinone derivatives (Cullen 1966). Furthermore, photodegradation of tetracycline has also been reported to result in the formation of a single oxygen atom (Frost 1971; Orentreich 1961). Tetracycline‐mediated reactive species have been reported to cause photohemolysis of erythrocytes and initiate damage to ribosomes (Davies et al. 1979; Green and Hill 1984). Reactive species, including hydroxyl radicals and singlet oxygen, can damage biomolecules within the cell and cause chain breaks in DNA (Epe et al. 1989). On peripheral blood lymphocytes, tetracycline induces cytogenetic damage, as evidenced by chromosome breakage and loss (Aydemir et al. 2005). The formation of DNA lesions has been demonstrated to block the processes of genome replication and transcription. Unrepaired DNA damage can result in the formation of mutations or lethal mutations, which may endanger the survival of the cell or organism or cause large‐scale genome errors. The analysis of DNA damage analyses offers significant insights into the early biological effects of exposure to harmful chemicals.

*D. magna* is an important model organism in the fields of ecotoxicology and genotoxicology research since it has a brief life cycle, straightforward cultivation, and exposure to a multitude of toxicants across diverse aquatic environments (Ebert 2005; Scheibener et al. 2021). The short life cycle of the organism offers a unique opportunity to observe and test the effects of toxic agents over multiple generations, as well as to examine the response of organisms at the DNA and individual levels. Its many behavioral and physiological parameters such as swimming speed, frequency of jumping, heart rate, swallowing rate, feeding rate, oxygen consumption, and thoracic limb activity are utilized to assess the toxicity of a specific substance. The utilization of these parameters confers the notable advantage of enabling the observation of nonlethal effects caused by low drug concentrations, which would otherwise remain undetectable through the application of OECD (Organization for Economic Co‐operation and Development) (OECD 2012) tests (Halligan and Keightley 2009; Jonczyk and Gilron 2005). It is, indeed, one of the most widely used model organisms for the assessment of the toxicity of a wide range of pharmaceuticals including antibiotics, anticancer drugs, antidepressants, anti‐inflammatory drugs, beta‐blockers, lipid‐regulating agents, and even naturally occurring radioactive elements (Halligan and Keightley 2009; Jonczyk and Gilron 2005).

While the phenotypic impacts of pharmaceuticals including tetracycline on aquatic biota are increasingly documented, the underlying mechanistic links between genomic instability and population‐level decline remain poorly understood. This study addresses this gap by testing the central hypothesis that tetracycline exposure compromises *D. magna* population sustainability via genotoxic mechanisms. In particular, we investigate that acute antibiotic stress induces primary DNA damage in the parental generation, which ultimately propagates as inherited fitness deficits in subsequent lineages. To evaluate this, we quantified the relationship between acute tetracycline exposure (25–400 mg/L over 24–72 h) and initial DNA damage alongside parental growth rates. Crucially, we tracked the persistence of genotoxic damage and their downstream consequences on reproductive fitness across unexposed offspring generations. By mapping molecular damage directly to multigenerational population growth rates, this work establishes whether transient genotoxic stress can precipitate long‐term population instability.

## 2 Materials and Methods

### 2.1 Culture Conditions

The life cycle of *D. magna* lasts 21 days. Accordingly, the experiment was repeated at the end of 21 days when new offspring were desired to be produced. The cultivation of *D. magna* was conducted within glass rectangular aquaria, with dimensions of 40 × 27 × 16 cm and a volume of 15 L. A filtration system was used within the aquarium to facilitate optimal growth and reproduction. In order to facilitate the optimal conditions for the healthy reproduction of *D. magna*, it was necessary to maintain environmental conditions of 21°C and a photoperiod at a natural level (16 h of daylight and 8 h of darkness). *D. magna* were fed when the water was clear, and the culture medium was replaced on a biweekly basis to encourage the production of new offspring (Gürbüz and Önalan 1998; Ersoy et al. 2007). *D. magna* were introduced to the culture water at a density of 2 × 10^3^ individuals per liter. An alternative method involved the arrangement of *D. magna* cultures in 100 mL glass jars containing 100 mL of culture media with 10 daphnids. A phytoplankton mixture was added to the culture jars daily basis, with an approximate concentration of 2 × 10^5^ cells/mL in order to feed *D. magna* (Wollenberger et al. 2000; Ersoy et al. 2007; Pellegri et al. 2014). This phytoplankton mixture comprised a variety of species including *Ankistrodesmus falcatus* , *Chlorella ellipsoidea* , *Chlorella pyrenoidosa* , *Scenedesmus ovalternus*. The density of the mixture was determined to be 56.33 × 10^6^ cells/mL (Gürbüz and Önalan 1998; Ersoy et al. 2007).

### 2.2 Preliminary Tests to Determine Tetracycline Exposure Concentrations

**FIGURE 1:**
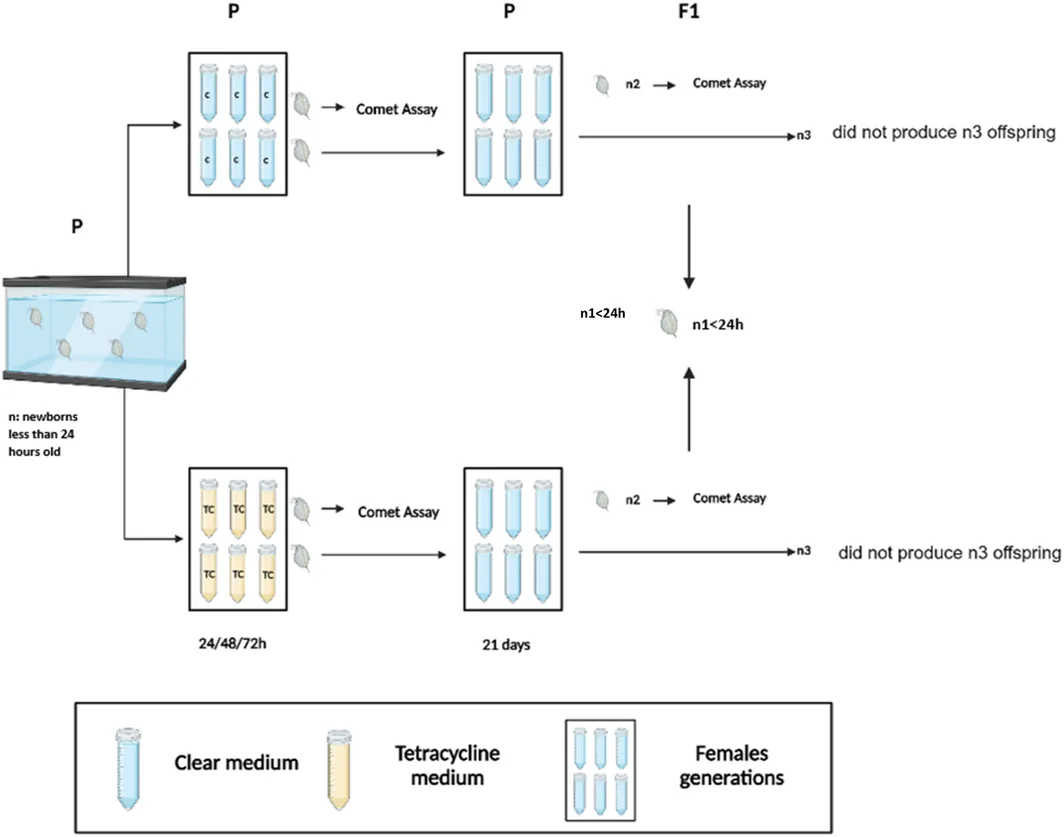
Schematic representation of the experimental design of the experiment. n1: newborns are less than 24 h old; n2 and n3: rood generations; c: control; TC: daphnids exposed to 25, 50, 100, 200, and 400 mg/L tetracycline for 24, 48, and 72 h (BioRender 2024).

The parental generation was subjected to a 21‐day exposure test, after which survival and reproductive success were subsequently evaluated. Tetracycline was administered at concentrations ranging from 25 to 400 mg/L for 24–72 h to determine the lethal concentrations (Scaria et al. 2021; Xie et al. 2011). A total of 30 pupae younger than 24 h were exposed to a range of tetracycline concentrations, including 25, 50, 100, 200, and 400 mg/L tetracycline concentrations for 24, 48, and 72 h (Figure 1).

During the 21‐day exposure test, the successive generations (P, F1) were transferred to culture media that was free of tetracycline and other contaminants. To investigate intergenerational effects, 20 offspring from four parental (P) females were collected to establish the first filial generation (F1). The neonates were categorized into three groups based on their age and the purpose of the experiment: n1: neonates less than 24 h old, who were excluded from the study to ensure developmental consistency, n2: 20–30 neonates aged 24–48 h, who were sampled for genotoxicity analysis, and n3: female neonates aged 48–72 h, who were intended for further culturing to produce the second filial generation (F2). The third newborn of each generation (n3), defined as newborns less than 24 h old, is considered the starting individual for the next generation (Reis et al. 2018). The experimental design entailed the performance of six biological replicates for each group.

Group culturing has been demonstrated to simulate more realistic population interactions and maintain sufficient biomass for the subsequent genotoxicity and intergenerational analyses (n1, n2, n3 groups). A total of 10 parental (P) individuals were cultivated in conjunction within a single vessel. While this methodology does not permit the tracking of individual offspring counts per female, it facilitates a more representative evaluation of the overall population's health and growth performance under tetracycline stress. In natural aquatic habitats, *Daphnia magna* populations are governed by continuous intra‐specific interactions, chemical signaling, and density‐dependent dynamics that significantly modulate their stress responses, and this approach was chosen to prioritize ecological realism over isolated monitoring.

### 2.3 Exposure Design

The exposure experiments were conducted in accordance with the experimental design proposed by Reis et al. (2018). To evaluate the effect of genotoxic damage on subsequent generations, individuals of the parental (P) generation were exposed to tetracycline concentrations of 25 and 50 mg/L for 24 h. Preliminary tests were conducted to determine lethal tetracycline exposure concentrations, and 25 and 50 mg/L were selected. The exposure conditions followed the same scheme as in Figure 1. To evaluate reproductive output, neonates produced by the P generation were collected and counted every 24 h throughout the observation period. Following each count, the P generation was transferred to fresh medium to maintain standardized conditions and prevent overcrowding. Reproductive success is presented as the (daily) number of offspring per female. The experiments were performed as six biological replicates. The control groups were cultivated under standard conditions for the duration of the experiment.

The parental generation (P) was exposed to tetracycline for 24, 48, and 72 h. Six parental females were sampled for the purposes of genotoxicity analysis. Additionally, 20 offspring from four of the parental females were collected and cultured (F1 generation). Newborns less than 24 h old (n1) were excluded from the study. 24–48 h old 20–30 newborns (n2) were sampled for genotoxicity analysis, and 48–72 h old females (n3) were collected and cultured (F2).

### 2.4 Individual Fitness

After 21 days of exposure to tetracycline, the body length of each female individual (P, F1) was measured from the crown of the head to the beginning of the caudal spine (the maximum body length). Each female individual was photographed using a light microscope (Dmi8, Leica), and subsequent measurements of body length were taken from the micrographs using the LAS X program (Reis et al. 2018).

### 2.5 Population Growth Parameters

To gain insight into the effect of tetracycline exposure on population growth, the approach proposed by Reis et al. (2018) was employed. However, the experiment was unable to proceed to the F2 generation due to a significant negative effect of tetracycline on population growth. The third‐born offspring (n3) of the first generation (F1) were found to be incapable of producing offspring as a consequence of the effects of tetracycline, and subsequently perished. In any case, the females were kept in isolation for a period of 21 days. To ascertain the survival rates of the parents and the reproduction success of the offspring, the number of surviving adults and juveniles, the number of offspring produced per individual, and the total number of offspring produced per individual were recorded.

### 2.6 Evaluation of DNA Damage

To determine the genotoxic effects of tetracycline treatment on *D. magna* , alkaline SCGE (single cell gel electrophoresis) was performed (Park and Choi 2007). About 50 organisms were mechanically disrupted in PBS homogenization buffer (40 mM EDTA and 20% DMSO) using a glass–glass homogenizer. The resulting lysate was passed through a cell strainer filter to remove nondegradable parts of the organisms. The lysate was mixed with 1% (w/v) low melting agarose (LMPA) to final concentration of 0.5% (w/v) and added to slides coated with 0.5% (w/v) normal melting agarose (NMPA). One percent (w/v) LMPA was added to the slides. The coated slides were incubated in lysis solution at 4°C in the dark. The lysis solution was prepared to contain 2.5 M NaCl, 100 mM EDTA‐Na_2_, and 10 mM Tris base (pH 10.0). One percent Triton X‐100 and 10% DMSO were added freshly to the lysis solution. Slides were incubated in electrophoresis buffer (300 mM NaOH, 1 mM EDTA disodium salt; pH > 13) for 20 min in the dark and electrophoresis was performed at 24 V (300 mA) for 30 min. After neutralization (Tris; pH 7.5), the samples were kept in 96% ethanol for 20 min and then dried in an oven at 50°C for 30 min. After neutralization, slides were stained with 2 μg/mL EtBr and observed under florescence microscope (Dmi8, Leica). A minimum of three slides were prepared for each replicate, and 100 nuclei were blindly scored. Tail moment of DNA obtained by Comet assay were expressed as AU in the present study, which were evaluated by visual scoring of nuclei representing DNA damage. Nuclei were scored as 0, 1+, 2+, 3+, and 4+ by a blinded observer according to apparent relative proportion of DNA in the tail and head. Each counted nucleus was multiplied by its score, and total scores were expressed as arbitrary units (AU) (García et al. 2004; İşeri et al. 2013).

### 2.7 Statistical Analyses

All data are presented as mean ± standard error of the mean (SEM) values derived from a minimum of three replicates of the experimental setups. All statistical analyses were performed using the Prism 11.0.0 Software package (GraphPad Software, USA). The mean differences between the control and tetracycline exposure groups were statistically evaluated by two‐way ANOVA analysis at the *p* < 0.05 level. Post hoc Tukey analyses were performed to identify significant differences. All statistical analyses were performed on the basis of the raw data.

## 3 Results

### 3.1 Tetracycline Caused Decrease in the Survival and Population Growth in a Concentration Dependent Manner

**FIGURE 2:**
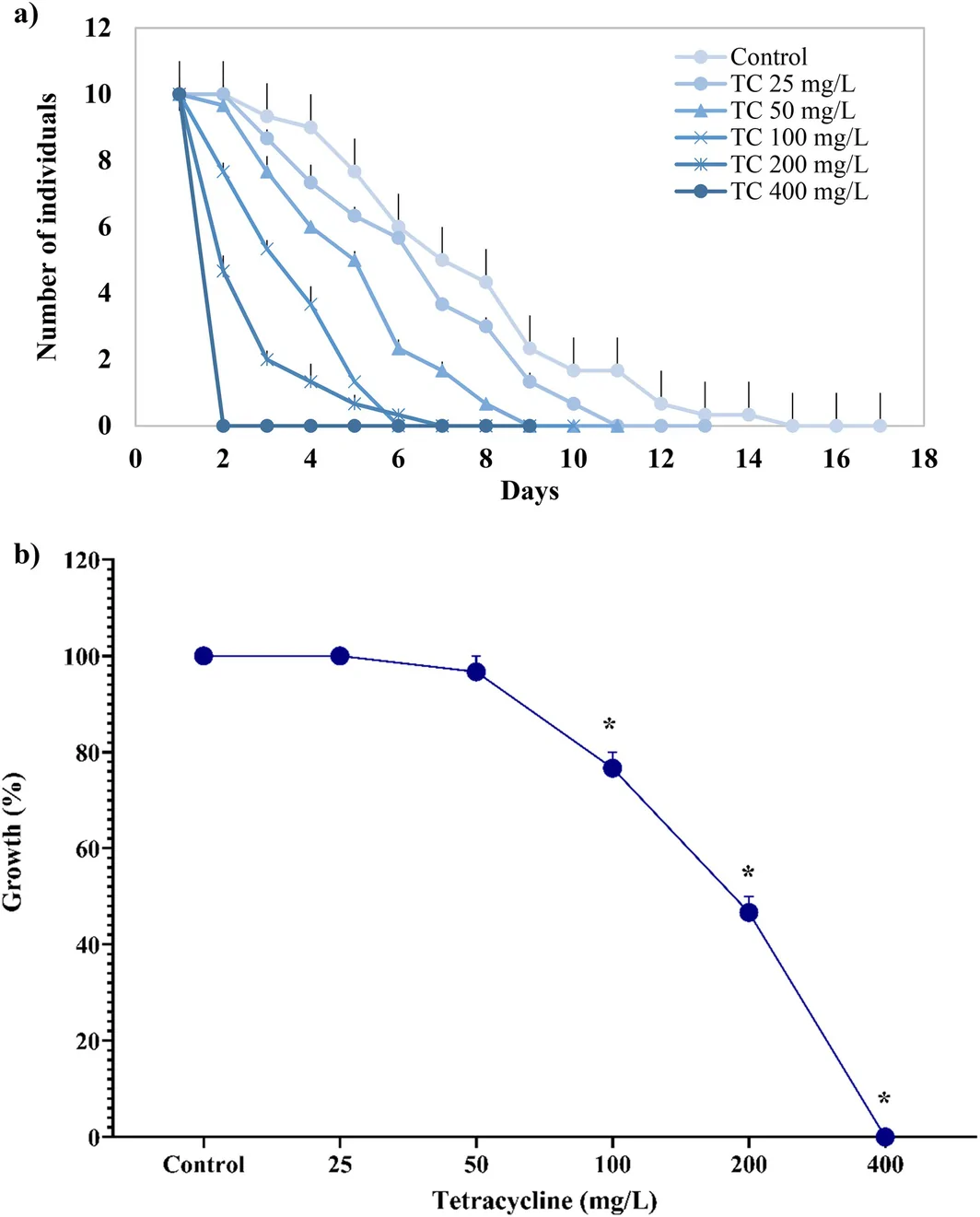
Toxicological profile of tetracycline in *D. magna*. (a) concentration‐dependent tetracycline exposure on the survival of P generation over time. (b) Acute impact of tetracycline on population growth rate over 24 h for the EC_50_ value. The data are expressed as the mean ± SEM. Asterisks represent statistical differences between the treatment and the control groups (*p* < 0.05).

In the ecotoxicological studies where *D. magna* is employed as a model organism, the survival rates of the number of individuals within 21 days and the reproduction success of the parents within the first 3 days are considered (Reis et al. 2018). However, the 21‐day continuous exposure could not be completed due to the high toxicity of tetracycline at the tested concentrations. It was observed that even at the lowest concentration of 25 mg/L, the parental generation (P) exhibited a maximum lifespan of only 18 days, thus preventing the completion of the standard 21‐day observation period. Consequently, the offspring genotoxicity test was designed to determine the test concentrations at which tetracycline would not be lethal and would nevertheless induce DNA damage. As illustrated to Figure 2a, the administration of 400 mg/L tetracycline resulted in the death of all individuals within 24 h, whereas exposure to 200 and 100 mg/L of tetracycline resulted in a significant reduction in population size after a period of 7 days. The survival rates of the subjects exposed to 50 mg/L of tetracycline were recorded as 9 days, with an additional 2 days observed in the subjects exposed to 25 mg/L of tetracycline.

The population growth of *D. magna* was found to be reduced following the administration of tetracycline (Figure 2b) after a period of 24 h. A concentration of 100 mg/L was found to result in a reduction in population growth of approximately 25%. The population growth was reduced to 47% at a tetracycline concentration of 200 mg/L, while no individuals survived at a concentration of 400 mg/L tetracycline. The half‐maximal effective concentration (EC_50_) is a well‐established endpoint in ecotoxicology that is utilized to define the critical effects caused by acute exposure (Iovine et al. 2024). It was determined based on the reduction in the number of individuals relative to the control group. To calculate this value, a nonlinear regression analysis was performed using a dose–response model. The raw data were fitted to a logarithmic trend line, and the EC_50_ was derived from the resulting equation where the response level equals 50% of the control's maximum. The EC_50_ was calculated as 154 mg/L.

In light of the effects of varying tetracycline concentrations on survival and population growth, it was observed that tetracycline concentrations of 25 and 50 mg/L caused less cytotoxicity within 72 h. As a result, it was considered appropriate to continue the testing at these concentrations.

### 3.2 Tetracycline Had Adverse Effects on Reproduction Success

To understand the effect on the population growth parameter, the survival of both adults and juveniles was evaluated over two generations, and reproductive failure was analyzed as a final endpoint. Despite the necessity of at least 20 offspring for generation transfer, the F2 generation did not reach adulthood and produce new offspring at the concentrations (25–400 mg/L) that were tested. Furthermore, a mortality rate of 100% was exhibited within 2 days. While this prevents the assessment of “trans‐generational” effects in the strictest sense, it highlights a critical reproductive “dead‐end” and the potent impact of these concentrations on the long‐term sustainability of the *D. magna* population. Consequently, since no viable offspring (F2) emerged from the F1 generation, the experimental procedures were continued only with the F1 generation.

**FIGURE 3:**
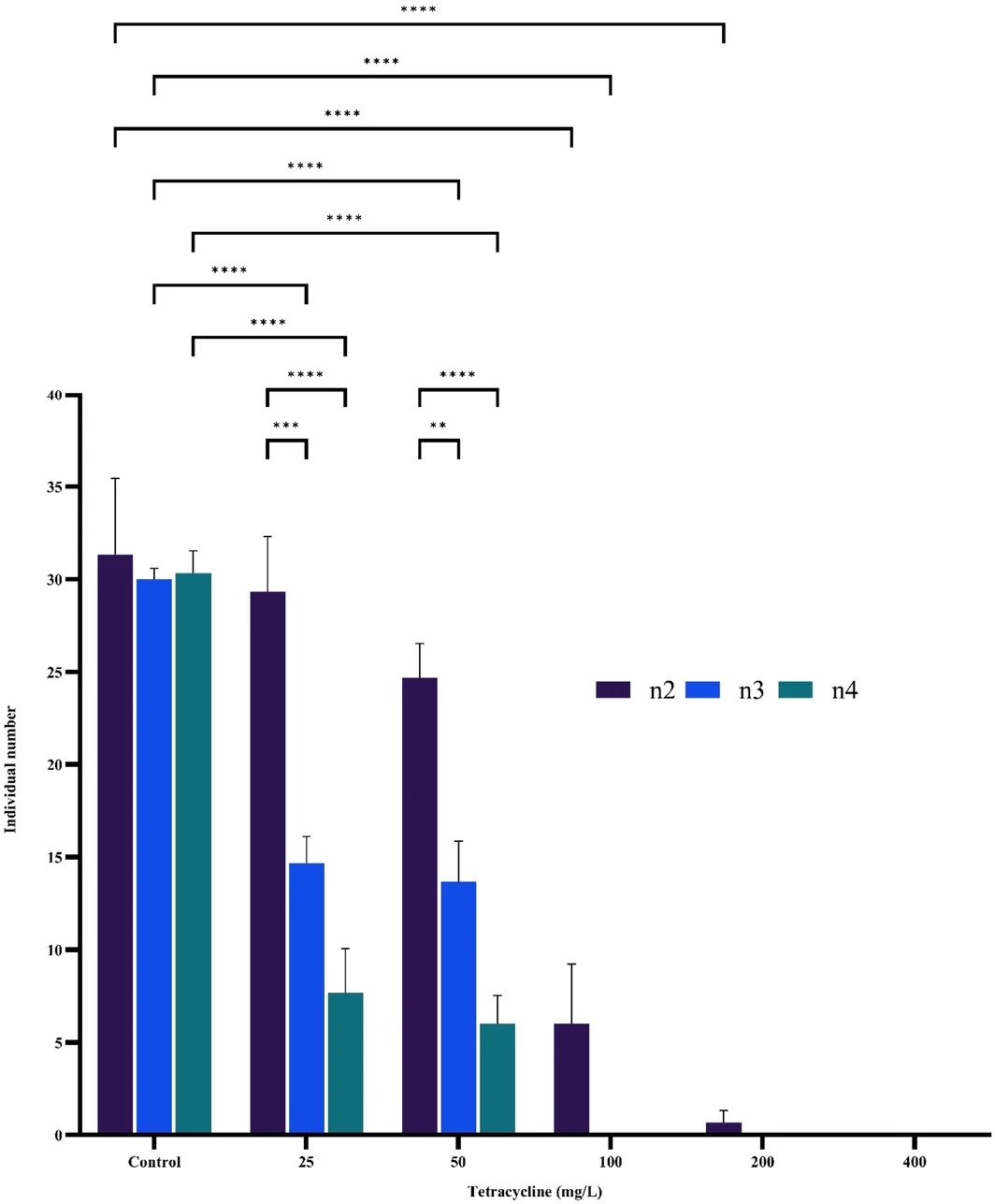
Reproductive success of F1 generation cohorts (n2, n3, n4) following parental exposure to tetracycline. Data illustrate the mean number of viable offspring produced per female in successive broods. Asterisks indicate significant statistical differences from the control group (*p* < 0.05). Data are expressed as mean ± SEM.

The findings of the study demonstrated a decline in the reproductive success of viable juvenile generations in response to elevated tetracycline concentrations (Figure 3). The data indicated that n2 of the F1 generation was affected by tetracycline exposure concentrations greater than 50 mg/L. On the other hand, a significant reduction in reproductive success was observed at a tetracycline concentration of 25 mg/L in the next generation. Furthermore, no n3 individuals or their offspring (n4) were observed in groups exposed to concentrations of tetracycline of 100 mg/L or higher. Accordingly, the administration of tetracycline resulted in a reduction in the survival and reproductive capacity of the parental groups, as well as an impairment in the growth and development of the offspring, which ultimately precluded their capacity to reproduce. In such cases of extreme toxic stress, traditional life‐history traits such as “timing of first brood” become less informative, as the primary biological response is the cessation of the reproductive cycle itself.

### 3.3 Tetracycline Caused Reduction in Individual Fitness for Intergenerations

**FIGURE 4:**
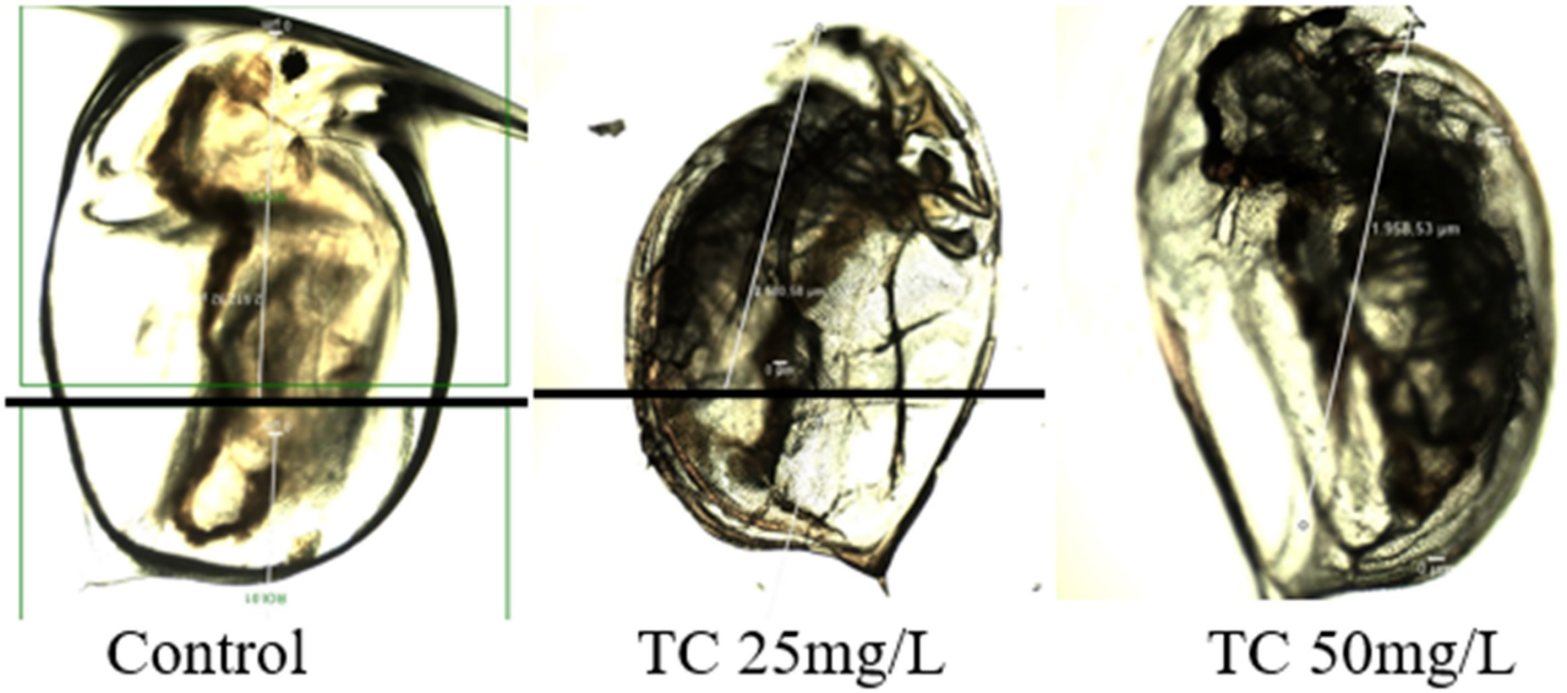
Representative micrographs of female *D. magna* (×50) (Dmi8, Leica).

In *D. magna* , body length is a well‐established and highly correlated indicator of reproductive potential. Larger females typically possess larger brood chambers and higher energy reserves. Therefore, the significant inhibition of body length observed in our study serves as a robust direct indicator of reduced individual fitness and future reproductive output (Enserink et al. 1990; Ebert 2005). In the present study, the impact of two different concentrations of tetracycline (25 and 50 mg/L) on the individual fitness of *D. magna* was evaluated. The duration of the exposure was 24–72 h, and the study was conducted over two generations. Meanwhile, female individuals were photographed using light microscopy to illustrate the distribution of tetracycline within the body structures and its accumulation (Figure 4). The circulatory systems of the tetracycline treated parents exhibited a darker pigmentation in comparison to the control groups.

**FIGURE 5:**
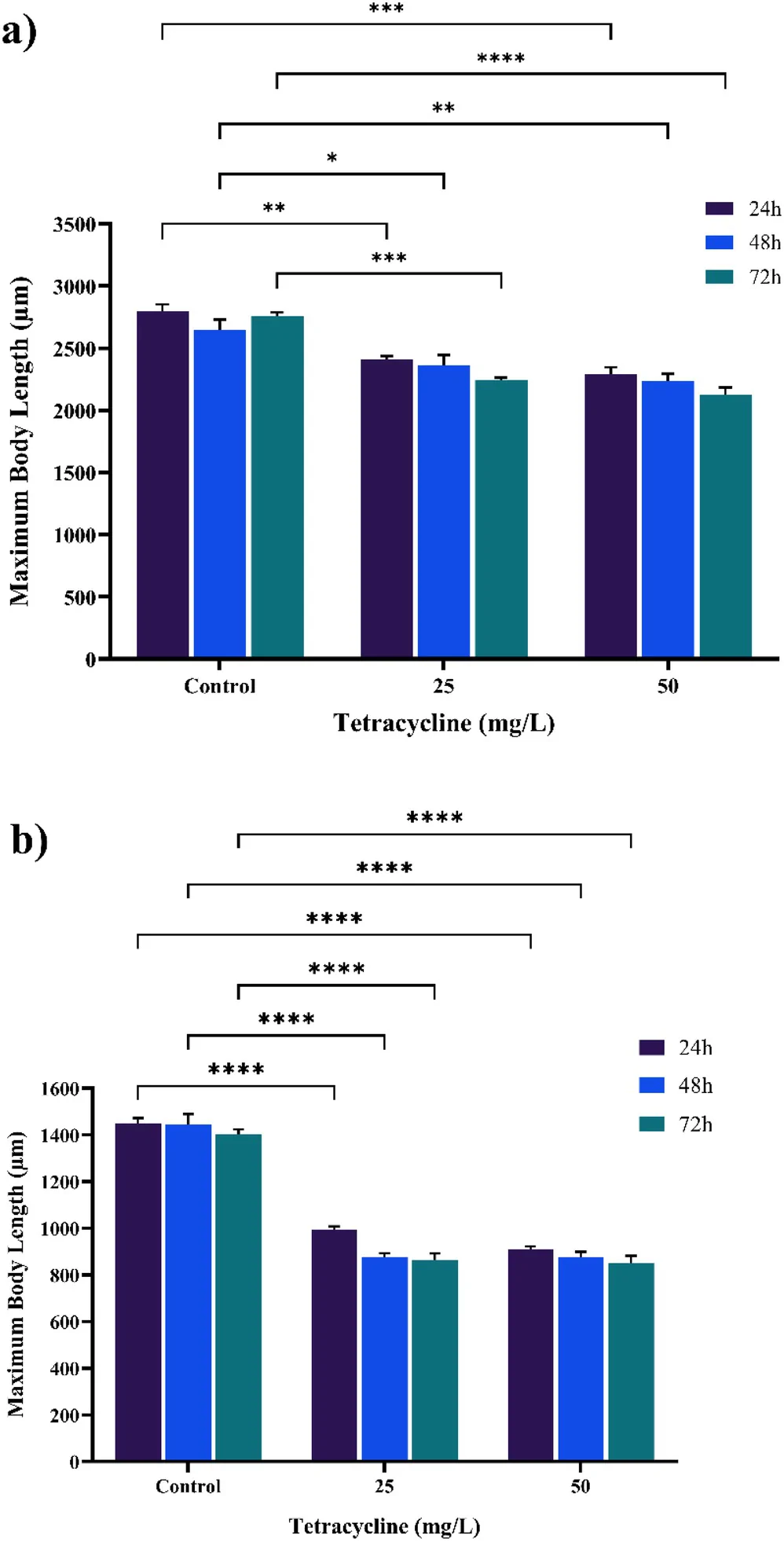
Maximum body lengths measured at different tetracycline concentrations and exposure times of the (a) parental generation and (b) first generation (F1) second neonates (n2). Body lengths were compared between the control group and the treatment group with one exposure time, as well as between the different exposure times within the treatment groups. The statistical differences between the control and treatment groups are represented by asterisks (*p* < 0.05). Data are expressed as mean ± SEM.

The maximum body length of *D. magna* was found to be significantly reduced in both generations (P and F1) exposed to tetracycline, in comparison to the control groups (Figure 5). The duration of exposure did not have any impact on the body length of the parental generation (Figure 5a). Furthermore, increased tetracycline concentration did not exert an influence on body length in the parental generation. The body length of the first generation (F1) second neonates (n2) exposed to 25 mg/L for 24 h did not differ from that of the controls. However, an increase in exposure time resulted in a reduction in body length (Figure 5b). The body lengths of the 48‐ and 72‐h exposure groups were found to be influenced by the concentration of tetracycline.

### 3.4 Tetracycline Induced Genotoxic Damage for Intergenerations

**FIGURE 6:**
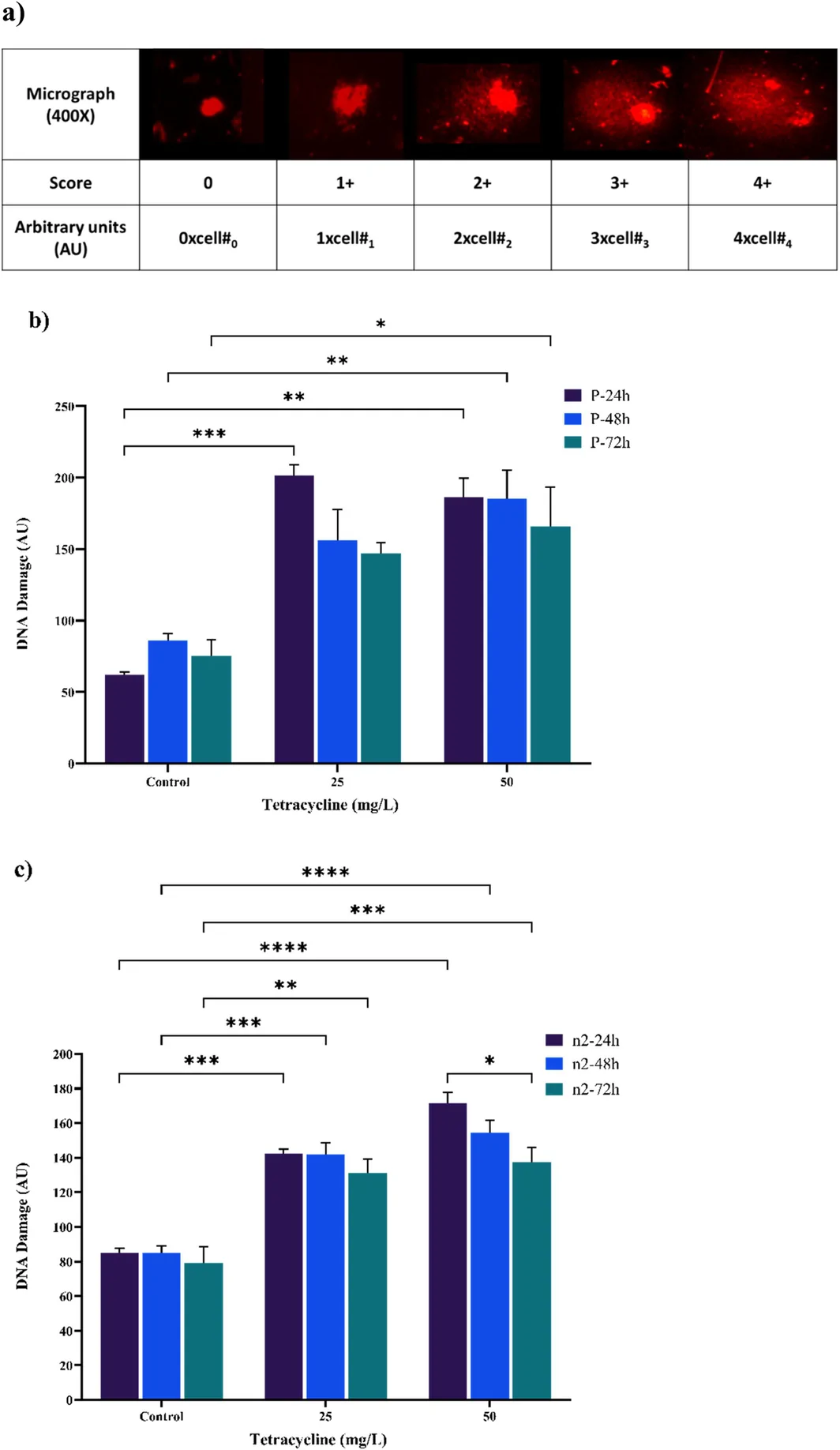
(a) Representative images and visual scoring of nuclei representing DNA damage. Nuclei were scored as 0, 1+, 2+, 3+, and 4+ according to relative proportion of DNA in the tail and head. Genotoxic damage induced by different tetracycline concentrations on (b) parent generations, and (c) first generation (F1) second neonates (n2). Total scores are expressed in arbitrary units (AU). The statistical differences between the control and treatment groups are represented by asterisks (*p* < 0.05). Data are expressed as mean ± SEM.

In the alkaline SCGE assay, DNA fragments move at different speeds in the agarose layers depending on their molecular weight depending on the degree of damage. In an alkaline environment, the chromatin structure is opened, and the DNA is partially denatured. Due to the action of genotoxic factors, DNA fragments of different molecular weights are formed due to breaks in the single and/or double strand of DNA. This results in images similar in the form of a comet (Figure 6a). Depending on the degree of damage, i.e., DNA fragments of different sizes and/or loosening of the supercoil structure, a circular “head” and a scattered “tail” image occur.

Comet analysis images are graded from 0 to 4 according to DNA damage levels, and the visual scoring is based on the evaluation of the amount of DNA that is relatively in the head or in the tail (Figure 6a) (García et al. 2004; Yurtcu et al. 2011). The amount of DNA is evaluated on the basis of a visual assessment of the density of fluorescence.

Given the lethal effects of tetracycline, the trans‐generational exposure test was not continued for three generations (for F2 and F3). The test was conducted on the offspring of F1 born between 48 and 72 h (n2) and the parental (P) generation, with the objective of testing for DNA damage. It was found that 24–72 h of exposure to 25 and 50 mg/L tetracycline resulted in DNA damage in *D. magna* (Figure 6). Tetracycline was found to cause genotoxic damage to the parent (P) generations at both concentrations (25 and 50 mg/L) and at all exposure times (Figure 6b), irrespective of concentration and exposure time. Similarly, exposure to 25 and 50 mg/L of tetracycline resulted in significant genotoxic damage in the second neonates (n2) of the first generation (F1) (Figure 6c). It has been determined that the damage to the DNA was found to be independent of the exposure time. In the experimental groups, the same degree of damage was observed after varying exposure times (*p* > 0.05). A comparison of the DNA damage observed in the parental and F1 generation after 72 h of exposure revealed that the newborns exhibited a reduction in DNA damage at varying concentrations.

## 4 Discussion

*D. magna* exhibits a high degree of sensitivity to alterations in their surrounding environment. Typically, asexual reproduction occurs through clonal propagation, while sexual reproduction is initiated under conditions of deteriorating environmental quality (Lampert 2011). Environmental signals have been demonstrated to initiate rapid responses in the morphology, physiology, and even behavior of these organisms (Laforsch and Tollrian 2004; Miyakawa et al. 2010). We utilized group culturing rather than isolated individual exposures to prioritize ecological realism, capturing population‐density dynamics and resource competition. However, we acknowledge that this culturing approach introduces a trade‐off, as it precludes the tracking of individual reproductive outputs and specific maternal life‐history trajectories. Consequently, our individual fitness metrics represent population averages rather than individual‐specific variations. Given that our primary objective was to evaluate overall population sustainability, this design choice favors community‐level relevance over individual precision.

Previous studies have documented the acute and chronic toxic effects of tetracycline on *D. magna* . In the study conducted by Wollenberger et al. (2000) the acute toxic effects (at 24 and 48 h) of tetracycline and oxytetracycline were examined at concentrations ranging from 1 to 340 mg/L and 1 to 100 mg/L, respectively. The tested tetracycline concentrations demonstrated reproductive toxicity within an EC_50_ range of 45 mg/L. The cytotoxic and genotoxic effects of tetracycline were also demonstrated in terrestrial plant models. Xie et al. (2011) reported chromosomal aberrations in wheat at 5 mg/L and a decreased mitotic index at concentrations above 50 mg/L. Tetracycline concentrations ranging from 10 to 300 mg/L exerted an influence on the parameters of wheat germination, shoot height, and root length after a 72‐h exposure period. The present study was conducted with the objective of establishing a dose–response relationship by testing the short‐ and long‐term effect of tetracycline in a concentration range of 25–400 mg/L. At low, environmentally trace levels, organisms may deploy homeostatic or adaptive mechanisms (such as upregulated antioxidant production or baseline DNA repair) that mask the observation of underlying toxic potential. To establish whether tetracycline possesses a genotoxic mode of action in *D. magna* capable of cascading into population‐level damage, it is necessary to challenge acute adaptive mechanisms within our experimental model. The determination of the “genotoxic lowest observed effect concentration” is of crucial importance to derive safe “predicted no‐effect concentrations (PNEC)” for environmental pollutants. One way to calculate PNEC is to divide EC_50_ by an assessment factor, which ranges from 10 to 1000 and accounts for uncertainties in extrapolating from laboratory tests to ecosystems (Alves Miranda et al. 2025). In genotoxicity studies, higher concentrations are tested to overcome the natural DNA repair mechanisms and determine DNA damage even at sublethal concentrations where mortality and toxicity are not yet observed at different end‐points. Furthermore, research indicates that genotoxic and cytotoxic “no effect concentrations” may drop ×100 from P to F1 and F2 generations (Kim et al. 2012) if reproductivity is not affected. While environmentally relevant lower levels may trigger adaptive responses such as the elevation of DNA repair mechanisms that mask potential damage, higher concentrations reveal the underlying toxic effects. Our results showed that the 24‐h EC_50_ value of tetracycline was 154 mg/L (Figure 2b). However, prolonged exposure to tetracycline significantly reduced the survival of the parental generation. Considering the effects of varying tetracycline concentrations on survival and population growth, it was observed that tetracycline concentrations of 25 mg/L and 50 mg/L caused less cytotoxicity within 72 h. Concentrations of 25 mg/L and 50 mg/L represent roughly 16% and 32% of the EC_50_. These concentrations are consistent with the sublethal ranges used to observe genotoxic damage without immediate organism death or cytotoxicity (İşeri et al. 2011). Consequently, these concentrations were selected to ensure sublethal yet detectable physiological stress and ascertain their potential for causing DNA damage in subsequent generations.

It has been demonstrated that tetracycline‐mediated reactive species can cause photohemolysis of erythrocytes and damage ribosomes (Davies et al. 1979; Green and Hill 1984). It is established that reactive oxygen species (ROS), including hydroxyl radicals and singlet oxygen, can damage biomolecules within the cell and cause strand breaks in DNA (Epe et al. 1989). A study of peripheral blood lymphocytes showed that tetracycline causes cytogenetic damage, manifesting as chromosome breakage and chromosome loss (Aydemir et al. 2005). Such DNA lesions have the potential to block the replication and transcription of the genome. Unrepaired DNA damage can lead to the emergence of mutations or lethal mutations that threaten the survival of the cell or organism, or alternatively, it can give rise to large‐scale genomic errors. Assays for DNA damage provide important insights into the early biological effects of exposure to toxic chemicals. The Comet assay is a method of examining DNA obtained from a single cell and nucleus in thin agarose gel layers in an electrophoretic environment. In the absence of damage, supercoiled DNA exhibits a slow, circular movement within the nuclear structure, as observed under a microscope. Due to lesions such as single and/or double strand breaks, cross‐links and base damage, the movement of DNA whose supercoil structure is greatly opened and/or fragmented into different dimensions is dependent on the molecular weight of the fragments (İşeri et al. 2013; Yurtcu et al. 2011).

Thus far, we have performed Comet assay for different cell types and compounds (İşeri et al. 2011, 2013; Yurtcu et al. 2011). Test organism is an important parameter since cell debris may produce background noise in fluorescent staining producing “false” comet tails in image analysis. The use of *D. magna* , a multicellular organism with a chitinous exoskeleton, presents unique challenges for automated comet analysis, such as background interference from tissue debris and mechanical artifacts (Figure 6a). By utilizing blind visual scoring, we ensured that only high‐quality, representative nuclei were included, thereby increasing the signal‐to‐noise ratio of our genotoxicity data. Noise usually results in a high frequency of Score 1 comets across all samples including controls, which may be mistakenly interpreted as abundance of low level ssDNA breaks and affect the baseline of all analysis. Furthermore, an extensive amount of cell debris may be analyzed as a score of 4, which indicates a high level of DNA damage, particularly caused by dsDNA breaks. Visual scoring into five categories (0–4) is a well‐established, validated method that has been shown to be as sensitive and reliable as computerized analysis (García et al. 2004). It is particularly effective for identifying low levels of DNA damage that automated systems may sometimes misinterpret as background noise.

The majority of *D. magna* that reached adulthood exhibited pronounced effects from tetracycline. The second‐born offspring showed a reduction in genotoxic damage in comparison to the parental generation. These results support that DNA damage is transmitted across generations, but not completely. As the number of generations of newborn pups increases, the repair of DNA damage begins to occur, and its transmission to other generations decreases. In a study conducted by Reis et al. (2018), it was observed that DNA damage was not transmitted to the third generation. Additionally, it was noted that while DNA damage was transmitted from the parental generation to the first generation, it was not detected in subsequent generations. These results confirm the findings of the present study, thereby confirming that DNA damage is indeed transmitted across generations but that mechanisms become operational at a certain point.

Although the results of the individual fitness test on *D. magna* revealed a significant reduction in the maximum body length of *D. magna* exposed to tetracycline in both generations, in comparison to the control groups. A comparable outcome was observed in the study conducted by Reis et al. (2018) wherein the administration of uranium to *D. magna* resulted in a notable reduction in maximum body length, particularly in the parental (P) generation. In the present study, however, the decrease was less pronounced when compared to the parental generation (P). This can be attributed to the repair of DNA damage in the offspring generation, which may result in differing fitness levels between parental and offspring generations. However, this conclusion is interpretative and requires further experimental evidence.

Moreover, darker color of the circulatory system colors of the *D. magna* in the tetracycline‐treated groups led to the conclusion that the *D. magna* absorb tetracyclines and incorporate them into their circulatory systems. Tetracycline has been demonstrated to affect reproduction and hepatoxicity in mammals (Farombi et al. 2008; Asha et al. 2007; Kim et al. 2012), with this effect being related to the production of ROS induced by tetracycline (Kim et al. 2012). This is primarily because the degradation of tetracyclines is accelerated in the presence of light, moisture, and oxygen. The presence of light has been demonstrated to cause photodegradation of tetracyclines and their derivatives. This effect of light occurs when the wavelengths of light are absorbed by these compounds. Tetracyclines have been observed capable of absorbing light within the visible and ultraviolet regions of the electromagnetic spectrum. This process gives rise to the formation of free radicals, which subsequently undergo degradation. Tetracyclines that demonstrate a robust absorption of violet and blue light manifest a yellow coloration. The effect of blue‐violet light on color change is the most significant, followed by white light. The effects of light on tetracyclines have been observed to be a result of both sunlight and artificial light. As demonstrated in a preceding study, Athanassiadis et al. (2018) has demonstrated that long‐term exposure to light from fluorescent lamps has been shown to cause photodegradation.

The results of the present study suggest that exposure to tetracycline exerts an adverse impact on the population growth parameters of the species. This is evidenced by the fact that the survival, growth, and reproduction of *D. magna* were found to be negatively affected. Previously, Kim et al. (2012) evaluated eight tetracycline concentrations of 100, 200, 500, 1000, 2000, 5000, and 1000 mg/L and found that none of them caused any acute effects. The 10,000 mg/L tetracycline concentration was found to result in a significant decrease in the total number of newborns in the parental generation. The number of newborns of the second (F1) generation was found to decrease in response to tetracycline concentrations of 5000 and 10,000 mg/L. The results of this study indicate that *D. magna* showed a significant chronic impact of tetracycline in terms of fertility (Kim et al. 2012). The present study hypothesizes that the mutations, chromosomal losses, cytogenetic damage, and genomic errors caused by tetracyclines in DNA affect the growth and development of *D. magna* . Consequently, tetracycline was found to inhibit the growth of both parental (P) and newborn (F1) offspring. This is because damage at the genomic level has the capacity to impede the processes of replication and transcription. The absence or incorrect synthesis of proteins within the cell may have deleterious consequences for the cell including cell signaling pathways and development. Concurrently, tetracycline has the potential to induce substantial genomic alterations and mutations, which can ultimately prove fatal and jeopardize the survival of the organism or cell. For all these reasons, tetracycline inhibits the growth of *D. magna* , preventing further development at both the cellular and organism levels.

## 5 Conclusion

In conclusion, this study demonstrates that even at low concentrations, tetracycline induces DNA damage in *Daphnia magna* . Notably, the DNA damage observed in the offspring was directly caused by parental exposure. Although daphnids appear to experience less DNA damage, the associated physiological cost is significant. Exposure to tetracycline exerts a detrimental effect on key life history traits, including individual fitness, survival, growth, and reproduction. These findings indicate that the impact of tetracycline transcends the molecular level, manifesting as substantial stressors at both the individual and population levels. Although *D. magna* displays a degree of resilience, the continuous release of tetracycline into aquatic ecosystems has the potential to pose a significant threat by increasing both the concentration and duration of exposure for nontarget organisms in these ecosystems.

## Author Contributions

Ö.D.İ. and Ç.A.P. conceived and designed the project and experiments. B.C.Ö., B.T., and Z.K. performed experiments. B.C.Ö., Ö.D.İ., and Ç.A.P. analyzed data and wrote the manuscript. The final manuscript was read and approved by all authors.

## Funding

This study was supported by The Scientific and Technological Research Council of Türkiye (TUBİTAK) (1919B012101796).

## Conflicts of Interest

The authors declare no conflicts of interest.

## Data Availability

The data that support the findings of this study are available on request from the corresponding author. The data are not publicly available due to privacy or ethical restrictions.
